# Access to Orthopedic Care for Deaf Patients With Distal Radius Fractures

**DOI:** 10.1177/15589447261415647

**Published:** 2026-02-02

**Authors:** Sophia Sarang Shin Yin, Arezo Ahmadi, Yew Song Cheng, Lauren M. Shapiro

**Affiliations:** 1 University of California, San Francisco, USA

**Keywords:** appointment time, deaf patients, health equity, interpreter, sign language, health outcomes, time to appointment, distal radius fracture, ad hoc interpreters

## Abstract

**Background:**

This study aimed to determine whether deaf patients experience barriers to orthopedic care compared with hearing patients through evaluating time to appointment, appointment denial rates, and interpreter availability.

**Methods:**

Researchers called 132 randomly selected US orthopedic offices to request appointments for fictitious patients with distal radius fractures. Each office was called twice on the same weekday over different weeks—once for a hearing patient and once for a deaf patient communicating in American Sign Language (ASL). The primary outcome was time to appointment. Secondary analysis included provider type, ASL interpreter availability, interpreter modality, and requests for family interpretation. Differences in time to appointment with *P* values were determined using Wilcoxon signed-rank, Mann-Whitney *U*, and Kruskal-Wallis tests.

**Results:**

Data from 132 clinics (63 academic and 69 community/private practices) were analyzed. The time to appointment for patients across all regions, practices, and providers was 3.9 days. Deaf patients experienced significantly longer wait times for physician appointments (4.96 vs 3.32 days, *P* value: .0031). When considering all providers (physicians, nurse practitioners, and physician associates), deaf patients did not wait significantly longer (4.43 vs 3.38 days, *P* value: .06). Most offices (81.8%) offered interpreters, with academic institutions more likely to guarantee ASL interpretation (95.5%) than community/private practices (68.2%). Some offices (17.9%) requested family members interpret instead.

**Conclusions:**

Distal radius fractures are common, and evidence suggests prompt care results in better outcomes and quicker return-to-work time. This study demonstrates statistically but not necessarily clinically significant delays for deaf patients seeking surgical appointments with MDs and reliance on ad hoc interpreters.

## Introduction

Timely access to health care is essential for achieving favorable outcomes, particularly when addressing urgent injuries like distal radius fractures (DRF). Earlier DRF surgery is associated with improved patient outcomes, including superior patient-reported outcomes, better range of motion, less stiffness, and fewer postoperative complications. For instance, studies by Hooper et al,^1^ Yamashita et al,^2^ and Sirniö et al^3^ found that earlier DRF surgery resulted in superior Disabilities of the Arm, Shoulder, and Hand scores at 6 weeks, 4 and 12 weeks, and 2 years, respectively. Yamashita et al and Luangjarmekorn et al found that earlier fixation was linked to better short-term wrist motion, and Ashdown et al found an association with decreased finger and thumb stiffness.^2,4,5^ Delayed surgical intervention has also been shown to increase the risk of complications, such as fracture malunion and infection, as evidenced by Grier et al.^6^ Given the importance of timely orthopedic intervention for DRFs, it is crucial to understand potential disparities in access to care.

The clinical benefits of timely care are not equally accessible to all patient populations. Studies across specialties have shown that patients who communicate in spoken languages other than English experience delays in care and difficulty securing appointments.^7-10^ Patients who are deaf and hard-of-hearing (DHH) who primarily communicate in American Sign Language (ASL) experience similar difficulties.^11,12^ In the United States, an estimated 8 million people have severe-to-profound hearing loss, 500 000 of whom use ASL to communicate.^13,14^ In the literature, DHH patients have reported barriers to care and appointment delays, which affect health care outcomes and relationships with the health care system.^11,12^ For example, Schniedewind et al^15^ found that hearing patients were almost 2 times more likely to secure primary care and dentistry appointments than deaf patients (adjusted odds ratio: 1.88, 95% confidence interval, 1.27-2.79). This disparity in appointment access has yet to be evaluated in orthopedic care.

As such, this investigation aims to evaluate access to orthopedic care by comparing appointment availability for simulated deaf and hearing patients with DRFs. Specifically, we assess the time to appointment, appointment denial rates, and the availability of interpreters. The primary null hypothesis predicts no significant difference in time to outpatient orthopedic appointments between simulated patients who are deaf and simulated patients who are hearing. The secondary null hypothesis posits that both cohorts will receive new patient appointments at similar rates. Last, we evaluated access to ASL interpreters by collecting information on how frequently ASL interpreters are available, if interpreters are in-person or via video, and if the office requests that the deaf patient bring a family member to interpret.

## Materials and Methods

The online “Find an Orthopaedist” American Academy of Orthopaedic Surgeons search tool was used to create a list of all orthopedic surgeons in all 50 states and Washington, DC who had identified the specialty of “Surgery of the Hand.” Providers who were listed as emeritus fellows, did not have a phone number listed, or cared only for pediatric patients were excluded.

An a priori power analysis was conducted to determine the sample size. No minimal clinically important difference in days for the treatment of DRFs has been standardized in the literature. Previous studies demonstrate that non-English-speaking patients face delays of 0.6 to 15 days for appointments compared with English-speaking patients.^7,9,10^ To conduct an a priori analysis for this study, we generated a clinically meaningful difference based on differences in appointment times reported in the literature, in addition to guidelines about DRF management from the British Orthopaedic Association, which suggest DRFs be evaluated within 3 days of injury.^7,9,10,16,17^ These sources collectively informed a clinically meaningful difference of 3 days. Based on the power analysis (power = 80%, α = .05, SD = 10), it was determined that 90 offices needed to be called. Accounting for an approximate 70% response rate, 132 offices were selected.^9^ The offices from the generated list were numbered, and a random number generator was used to select the offices of 132 orthopedic surgeons.

Two members of the research team were involved in calling offices. The investigators stated that they were calling to request an appointment with an orthopedic hand surgeon on behalf of their 55-year-old father, who had a DRF. Investigators called each office twice; one call was placed on behalf of a hearing patient, and one was made for a deaf patient who could communicate only in ASL. Before calling, the investigator flipped a coin to determine the patient’s preferred language for the first call. The same script was used and included the patient’s history, possible diagnosis of a distal radial fracture by urgent care through x-rays, private insurance plan information, and local address. Researchers asked for the soonest available appointment time. When calling on behalf of the deaf patient, the researcher requested an ASL interpreter and inquired whether the interpreter would be in-person or on video. Researchers called on the same weekday of different weeks (ie, if the first call was placed on Monday morning, the second would also be on Monday morning). The caller spoke English in both scenarios.

The researchers noted if an appointment was offered, the appointment date, and with whom (physicians [MD], nurse practitioners, and physician associates) it was offered. Clinics were categorized as either academic (affiliated with an academic hospital) or community/private practice. Clinics were categorized based on region (West, Northeast, South, or Midwest) using the US Census Bureau definitions. For simulated deaf patients, ASL interpreter availability, interpreter type (in-person or video), and requests for families to interpret were documented. Reasons for failed appointment scheduling were noted. Any appointments that were scheduled were promptly canceled the same day.

### Statistical Analysis

Descriptive statistics were used to summarize and report results. Wilcoxon signed-rank test and Mann-Whitney *U* tests were used as the data were not normally distributed. These tests were used to analyze statistical differences in time to appointment between hearing and deaf patients and subgroup analyses (eg, provider type and hospital type) for continuous variables. Researchers used a Kruskal-Wallis test to statistically compare the time to appointment for multiple groups (eg, region). *P* values less than .05 were classified as statistically significant. Chi-square tests were used for categorical variables (eg, access to appointment). Unsuccessful appointments were categorized based on whether the cause was related to language or interpreter services, or due to other factors (eg, insurance). For simulated patients who were deaf, we calculated the percentage of offices that offered an interpreter, the distribution of interpreter type (in-person vs video), and the percentage of offices that requested a family member to interpret.

## Results

Researchers identified and called a total of 132 clinics. This represented 40 states; 63 (47.7%) clinics were academic, and 59 (52.3%) were community clinics or private practices.

### Time to Appointment

**Figure 1.**
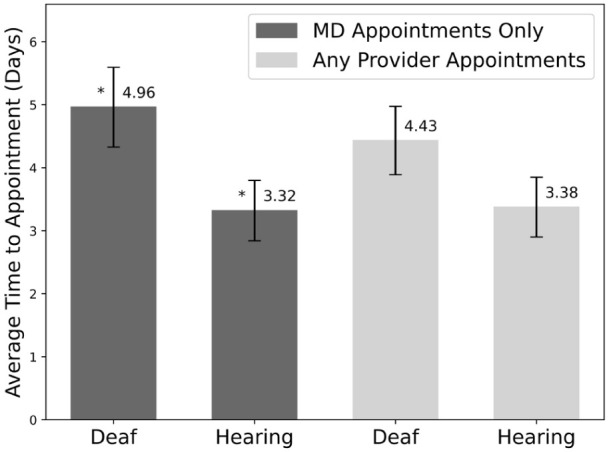
Average time to appointment in days for deaf and hearing patients for physician appointments only and for all provider appointments. Error bars show the standard error of the mean. Starred values are statistically significant (*P* value < .05).

The time to appointment for patients across all regions, practices, and providers was 3.9 days (SD: 4.80). Simulated deaf patients experienced statistically significantly longer wait times for physician appointments than simulated hearing patients (4.96 ± 0.63 days (standard error of the mean [SEM]) vs 3.32 ± 0.48 days [SEM], *P* value: .0066), as seen in Figure 1. Although the 1.64-day delay that deaf patients faced was statistically significant, it did not meet the originally defined clinical significance. For appointments with any provider, deaf patients had a longer but statistically insignificant wait time (4.43 ± 0.54 days [SEM] vs 3.38 ± 0.47 [SEM], *P* value: .067) (Figure 1).

**Figure 2.**
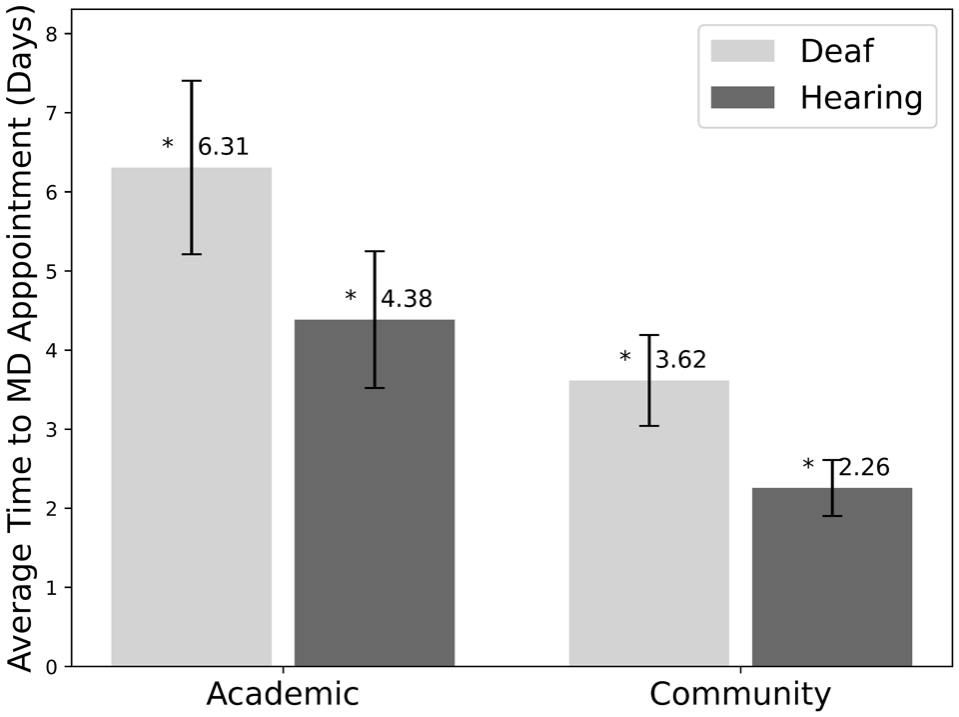
Average time to physician appointment in days for deaf versus hearing patients by hospital type (academic and community). Error bars show the standard error of the mean. Starred values are statistically significant (*P* value < .05).

**Figure 3.**
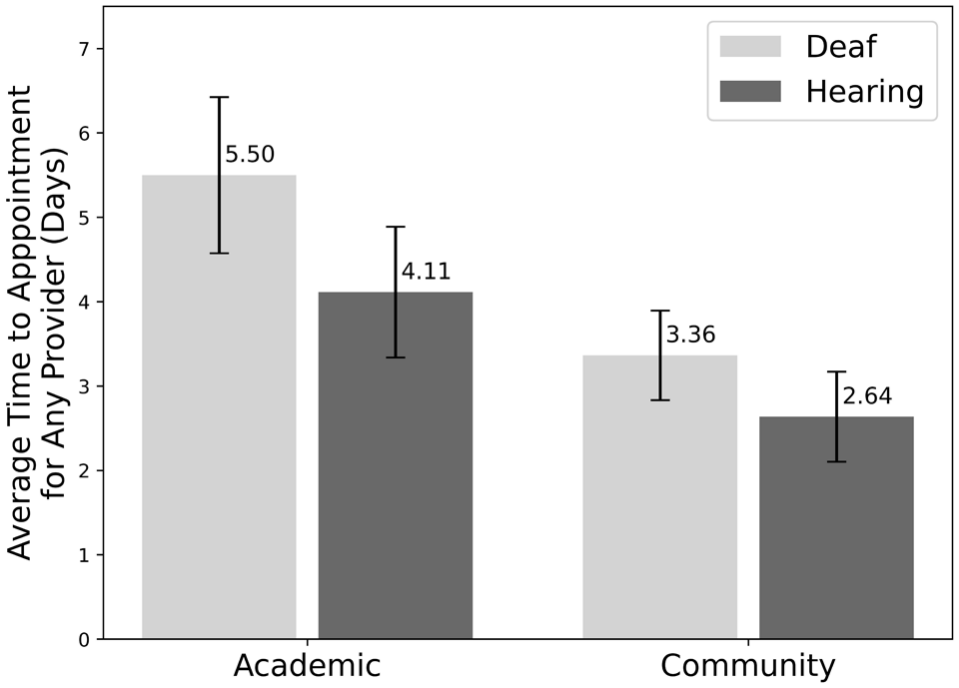
Average time to any provider appointment in days for deaf versus hearing patients by hospital type. Error bars show the standard error of the mean.

As seen in Figure 2, deaf patients waited significantly longer for MD appointments at both academic hospitals (6.31 ± 1.10 days [SEM] vs 4.38 ± 0.87 days [SEM], *P* value: .032) and community hospitals and private practices (3.62 ± 0.57 days [SEM] vs 2.26 ± 0.35 days [SEM], *P* value: .043). For appointments with any provider, the difference between deaf and hearing patients was not statistically significant, as shown in Figure 3 (*P* value: .23 for academic, *P* value: .12 for community/private practice).

**Figure 4.**
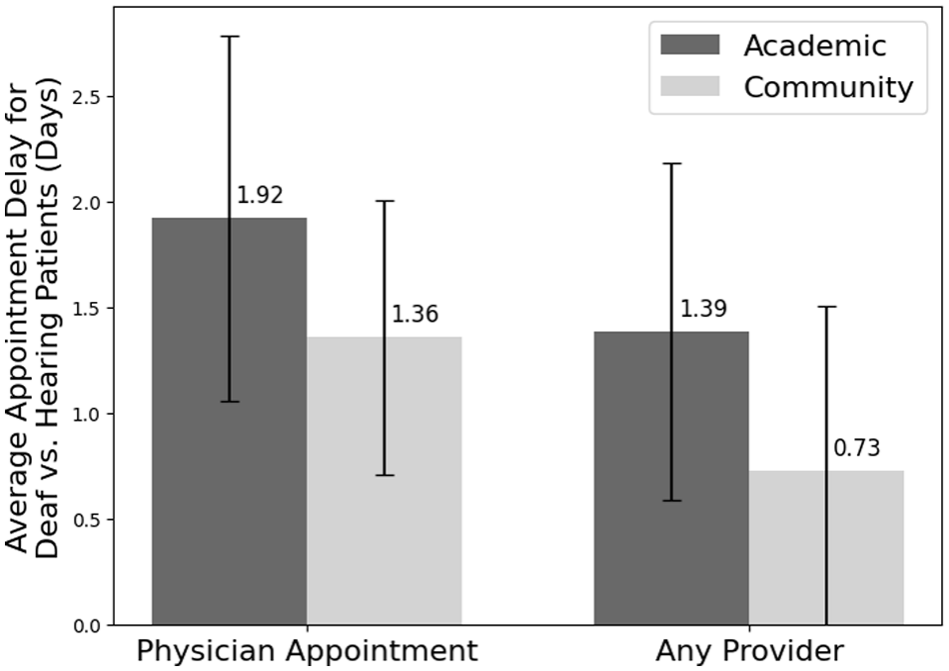
Average delay in appointment for deaf versus hearing patients by hospital type (days). Error bars show the standard error of the mean.

**Figure 5.**
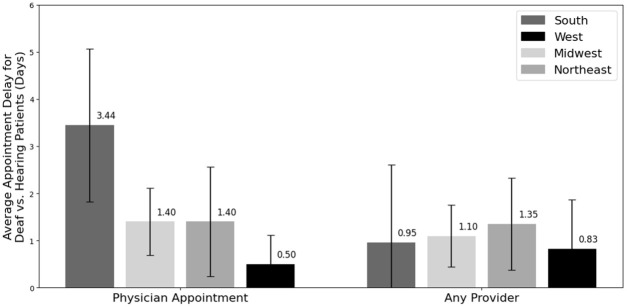
Average delay in appointment for deaf versus hearing patients by region (days). Error bars show the standard error of the mean.

On average, deaf patients experienced a 1.64-day delay for MD appointments (SEM: 0.54) and a 1.05-day delay for all provider appointments (SEM: 0.56). The delay that deaf patients faced was not significantly different at different hospital types for MD appointments (*P* value: .75) or for all provider appointments (*P* value: .71) (Figure 4). The same was true by region for MD appointments (*P* value: .47) and all provider appointments (*P* value: .61) (Figure 5).

### Access to Appointment

There was no difference in access to an appointment with an orthopedic provider between simulated hearing and simulated deaf patients (70.5% and 72.7%, respectively, *P* value = .78). Of the appointment denials, 38.7% of offices required additional insurance information (eg, insurance member identification), 22.6% required a referral (eg, from an urgent care physician), and 19.4% required radiographs or urgent care notes.

### Access to a Qualified Interpreter

**Figure 6.**
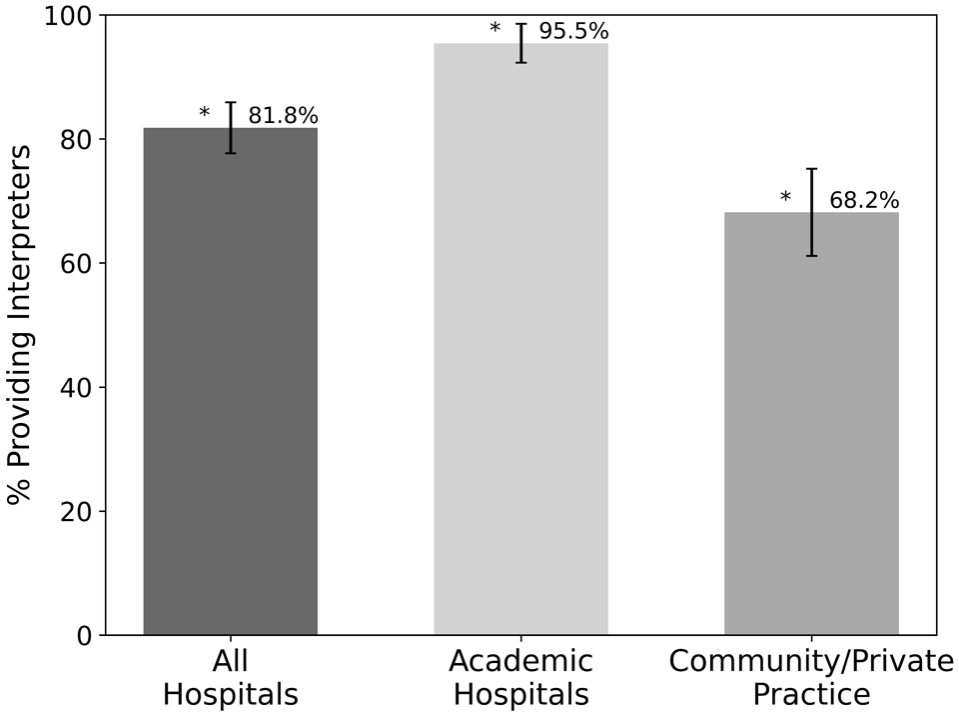
Percentages of hospitals or clinics that provided American Sign Language interpretation by hospital type. Error bars show standard deviation. Starred values are statistically significant (*P* value < .05).

Most offices (81.8%) provided an ASL interpreter on request or asked whether an ASL interpreter was needed (Figure 6). Regarding interpreters, 95.5% of academic hospitals provided interpreters versus 68.2% of community hospitals, which was statistically significant (*P* value: .0024) (Figure 6). There were no significant differences in interpreter availability across regions (*P* value: .265).

In 17.2% of cases, the office requested that or asked whether the patient could bring a family member to interpret. Of the offices that agreed to provide interpreters, 48.1% of offices guaranteed an in-person interpreter, 32.7% of offices offered video interpretation, 13.5% of offices guaranteed video interpretation with in-person interpretation pending subject to availability, and 5.8% of offices did not know the type of interpretation available.

## Discussion

In this cross-sectional study, deaf patients waited statistically significantly but not clinically significantly longer than hearing patients for physician appointments, but not for all provider appointments. Furthermore, interpreters were not always available for deaf patients, as almost a fifth of all offices did not provide sign language interpreters. These disparities in time to appointment and interpreter access across orthopedic practices raise concerns about accessibility, equity, and health care outcomes for deaf patients.

Health care outcomes for deaf patients may be negatively impacted as a result of delayed physician appointment times and interpreter inaccessibility. While deaf patients may have equal access to other providers (nurse practitioners, etc), physicians often ultimately decide and perform definitive treatment; thus, prolonged access to physicians can lead to delays in definitive care. Delays in surgery for DRFs may lead to worse outcomes, including increased stiffness, increased intraoperative and postoperative complications, and worse patient-reported outcomes.^2,4,5^ From an equity perspective, this difference is important as delayed access and poorer outcomes for one patient group can exacerbate health disparities and compromise overall well-being compared with those with timely access.

Although the difference between hearing and deaf patients is 1.64 days and did not reach the originally defined clinical significance of three days, the disparities in wait times are relevant from a health equity perspective. The British Orthopaedic Association recommends that DRF patients be assessed within 72 hours of presentation to reduce risk of complications.^16,17^ Yet, deaf patients will require interpretation services at every visit (e.g., pre-operative and peri-operative appointments) during their treatment timeline, suggesting their ultimate time to surgery may be compounded by more delays. In addition, in this study, the fictitious patients had United Healthcare PPO insurance plans instead of Medicare or Medicaid, which may have increased the likelihood of successful appointment booking. This insurance plan was chosen to remove possible barriers to securing appointments. However, deaf individuals are more likely to be on public insurance, and Medicaid patients experience more difficulty booking appointments.^18,19^ Not only may insurance status affect wait times, but interpreter needs can delay appointments if more time is required to secure interpreters. Thus, deaf patients experience multiple intersectional barriers that may exacerbate delayed access to care. Furthermore, although not originally defined as clinically significant, a delay of 1.64 days, compounded by other delays, results in losing days at work and time caring for one’s family and community.

Delayed appointment times for deaf patients may be explained by challenges in arranging interpreting services. For example, at least 5 offices mentioned interpreters had to be scheduled 1 to 2 weeks in advance. Additionally, 7 offices mentioned a delay in interpreter scheduling and stated they were suggesting a later appointment time than would be offered to a hearing patient to accommodate interpreter services. Notably, this study did not find a difference in the percentage of successfully booked appointments between hearing and deaf patients (70.5% and 72.7%, respectively), which differs from the findings of Schniedewind et al,^15^ which found hearing patients were twice as likely to secure primary care and dentistry appointments compared with deaf patients. This may be explained by the fact that our study classified visits that did not offer interpreters as secured appointments, whereas Schniedewind et al^15^ recognized only visits in which an interpreter was offered. Furthermore, unlike in Schniedewind et al, the researchers could not evaluate whether interpreters were used. Overall, however, the findings of delayed appointment times raise important concerns about access, particularly for conditions requiring urgent care.

In addition to delayed appointment times, this study found that a substantial portion of offices did not provide ASL interpreters, which may ultimately affect care outcomes.^20^ Instead, offices stated it was the patient’s responsibility to provide an ASL interpreter, whether through their insurance or out of pocket. Approximately 20% of offices also asked whether a family member could interpret. This limited ASL interpreter use may result in physicians relying on other communication methods, such as attempts at written communication, which can increase patient distress and frustration with health care services.^21^ In fact, at least 2 offices asked whether the patient and physician could write instead of providing an interpreter. This misunderstanding and disconnect from health care providers may result in mistrust surrounding health care services, which has been associated with delayed care and negatively impacts health care outcomes.^22,23^ Previous studies have found similar trends in other non-English-speaking groups, such as Spanish-speaking patients experiencing reduced interpreter access and being asked to use ad hoc interpreters.^7,9^ Such outcomes may explain the deaf community’s notably lower health literacy and higher rates of some chronic conditions, including obesity, uncontrolled hypertension, and mental health conditions.^24^

The right to access ASL interpretation for deaf patients is protected by the Americans with Disabilities Act (ADA) of 1990. The ADA is a federal law that prohibits any discrimination based on disability.^25^ Specifically, Title III of the ADA mandates “public accommodations” to provide “auxiliary aids and services,” which includes ASL interpreters for deaf individuals.^26^ Particularly, Title 28 of the Code of Federal Regulations, Section 115.216 states that “effective communication” with deaf people requires “interpreters who can interpret effectively, accurately, and impartially.” While family members may be able to converse with a patient, they may not possess the comprehensive knowledge of medical sign language needed to interpret “effectively” and “accurately.” Furthermore, relying on family members as interpreters can introduce bias and “partiality” due to their emotional involvement, which may compromise the objectivity of communication. Thus, to combat communication inaccessibility and comply with the ADA regulations, it is recommended that ASL interpreters be available at no cost to deaf patients.

The results should be understood in the context of their limitations. First, while this study was designed to have a hearing family member make an appointment on behalf of their deaf father, not every deaf patient has an individual to make such a call. Thus, this study may not be generalizable to all deaf patients and their level of health care accessibility. In addition, this study was designed to call offices and create new patient files, rather than mimic the more common process of patients being referred to surgeons by their primary care physician. Therefore, the results may overestimate the time needed to meet with an orthopedic surgeon.^27^ Moreover, the hearing investigator and fictitious patient did not attend the scheduled appointments; consequently, the study investigators could not check to see whether interpreters were actually used nor assess follow-through from offices claiming to provide interpretation services. Furthermore, all appointments were made by English-speaking individuals on the phone. This study did not include simulated non-English-speaking individuals, who may face additional barriers when scheduling appointments with English-speaking staff. Last, our study inferred a clinically meaningful difference for the evaluation of patients with DRFs based on literature and recommendations from the British Orthopaedic Association, as no minimal clinically important difference in number of days has been standardized in the literature.

This study found that deaf patients experience statistically significant but not clinically significant delays in appointment times with MDs compared with their hearing counterparts, as well as barriers to accessing interpreters. Future research could investigate the impact of other social determinants of health on communication access and health care outcomes. In addition, clinicians and hospital staff should improve access to interpreters at no cost to prevent delays in care for deaf patients. This study advocates for ADA policy recognition and follow-through to promote increased interpreter services and expanded health care accessibility for all.
